# Supplementary material for: Is there a role for neuregulin 4 in human nonalcoholic fatty liver disease?
Source: PLoS One. 2021 May 14;16(5):e0251822. doi: 10.1371/journal.pone.0251822 (PMC8121306; doi:10.1371/journal.pone.0251822)
Supplement: S1 Table — B: unstandardized regression coefficient, 95% CI: 95% confidence interval. The multivariable models include age, gender and presence of NAFLD as independent variables. Assumptions for linear regression were met since there were no influential outliers based on Cooks distance ≤ 0.184, and collinearity was met as indicated by variance inflation factor values ≤ 1.201. R square of the model was 0.054. (DOCX) [file pone.0251822.s001.docx]

**S1 Table: Multivariable regression analyses of Nrg4 levels in the total study population (n 108)**

| **Variable** | **Adjusted effects** | | |
| --- | --- | --- | --- |
|  | B | 95% CI | P-value |
| **Age**  **Gender (female vs. male)**  **Presence of NAFLD (yes vs. no)** | -0.202  -7.550  7.773 | -0.731; 0.326  -21.708; 6.608  -1.891; 17.357 | 0.449  0.293  0.114 |

B: unstandardized regression coefficient, 95% CI: 95% confidence interval. The multivariable models include age, gender and presence of NAFLD as independent variables. Assumptions for linear regression were met since there were no influential outliers based on Cooks distance ≤ 0.184, and collinearity was met as indicated by variance inflation factor values ≤ 1.201. R square of the model was 0.054.
