# Supplementary material for: Is there a role for neuregulin 4 in human nonalcoholic fatty liver disease?
Source: PLoS One. 2021 May 14;16(5):e0251822. doi: 10.1371/journal.pone.0251822 (PMC8121306; doi:10.1371/journal.pone.0251822)
Supplement: S2 Table — TNF- α, tumor necrosis factor alpha; IL-6, Interleukin 6; IFN-γ, interferon gamma. (DOCX) [file pone.0251822.s002.docx]

**S2 Table. Spearman’s rank correlations with Nrg4 and inflammatory markers in the total study population (n=108).**

|  | **Spearman’s rank (ρ)** | **p-value** |
| --- | --- | --- |
| **TNF-α** | -0.056 | 0.562 |
| **IL-6** | 0.152 | 0.117 |
| **IFN-γ** | 0.000 | 0.998 |

TNF- α, tumor necrosis factor alpha; IL-6, Interleukin 6; IFN-γ, interferon gamma.
