# Supplementary material for: Is there a role for neuregulin 4 in human nonalcoholic fatty liver disease?
Source: PLoS One. 2021 May 14;16(5):e0251822. doi: 10.1371/journal.pone.0251822 (PMC8121306; doi:10.1371/journal.pone.0251822)
Supplement: S3 Table — Values are presented as median (Q1; Q3) (skewed data) for continuous variables or number (% of group) for categorical variables. LDL-C, low-density lipoprotein cholesterol; HDL-C, high-density lipoprotein cholesterol; ALT, alanine transaminase; AST, aspartate transaminase; γ-GTP, gamma-glutamyltranspeptidase; AP, alkaline phosphatase. (DOCX) [file pone.0251822.s003.docx]

**S3 Table: Baseline characteristics patients used for microarray analysis liver and visceral fat tissue**

|  | **Obese, no NAFLD (n=6)** | **NAFL (n=4)** | **NASH (N=5)** |
| --- | --- | --- | --- |
| **Gender (female)** | 5 (83%) | 4 (100%) | 4 (80%) |
| **Age (years)** | 46 (43; 51) | 43 (34; 52) | 50 (46; 56) |
| **BMI (kg/m2)** | 51.2 (35.5; 53.6) | 48.3 (44.5; 54.8) | 40.8 (37.7; 48.9) |
| **Fasting Glucose** | 5.0 (6.1; 6.7) | 5.9 (5.1; 13.3) | 8.1 (6.4; 11.6) |
| **Total cholesterol (mmol/L)** | 5.19 (3.00; 5.50) | 5.14 (4.32; 6.43) | 4.74 (3.88; 6.05) |
| **LDL-C (mmol/L)** | 3.38 (3.00; 6.61) | 3.78 (2.52; 4.76) | 2.92 (2.27; 3.89) |
| **HDL-C (mmol/L)** | 1.01 (0.88; 1.28) | 1.21 (0.83; 1.21) | 0.91 (0.86; 1.16) |
| **Triglycerides (mmol/L)** | 1.81 (1.13; 2.50) | 1.51 (1.26; 1.72) | 1.71 (1.26; 3.01) |
| **ALT (U/L)** | 20 (16; 25) | 27 (17; 56) | 46 (19; 103) |
| **AST (U/L)** | 18 (16; 24) | 26 (17; 37) | 30 (19; 70) |
| **γ-GTP (U/L)** | 25 (13; 30) | 23 (19; 29) | 27 (19; 55) |
| **AP (U/L)** | 77 (57; 111) | 46 (42; 85) | 52 (46; 74) |

Values are presented as median (Q1; Q3) (skewed data) for continuous variables or number (% of group) for categorical variables. LDL-C, low-density lipoprotein cholesterol ; HDL-C, high-density lipoprotein cholesterol; ALT, alanine transaminase ; AST, aspartate transaminase; γ-GTP, gamma-glutamyltranspeptidase; AP, alkaline phosphatase.
