# Supplementary figures and images for: Is there a role for neuregulin 4 in human nonalcoholic fatty liver disease?
Source: PLoS One. 2021 May 14;16(5):e0251822. doi: 10.1371/journal.pone.0251822 (PMC8121306; doi:10.1371/journal.pone.0251822)

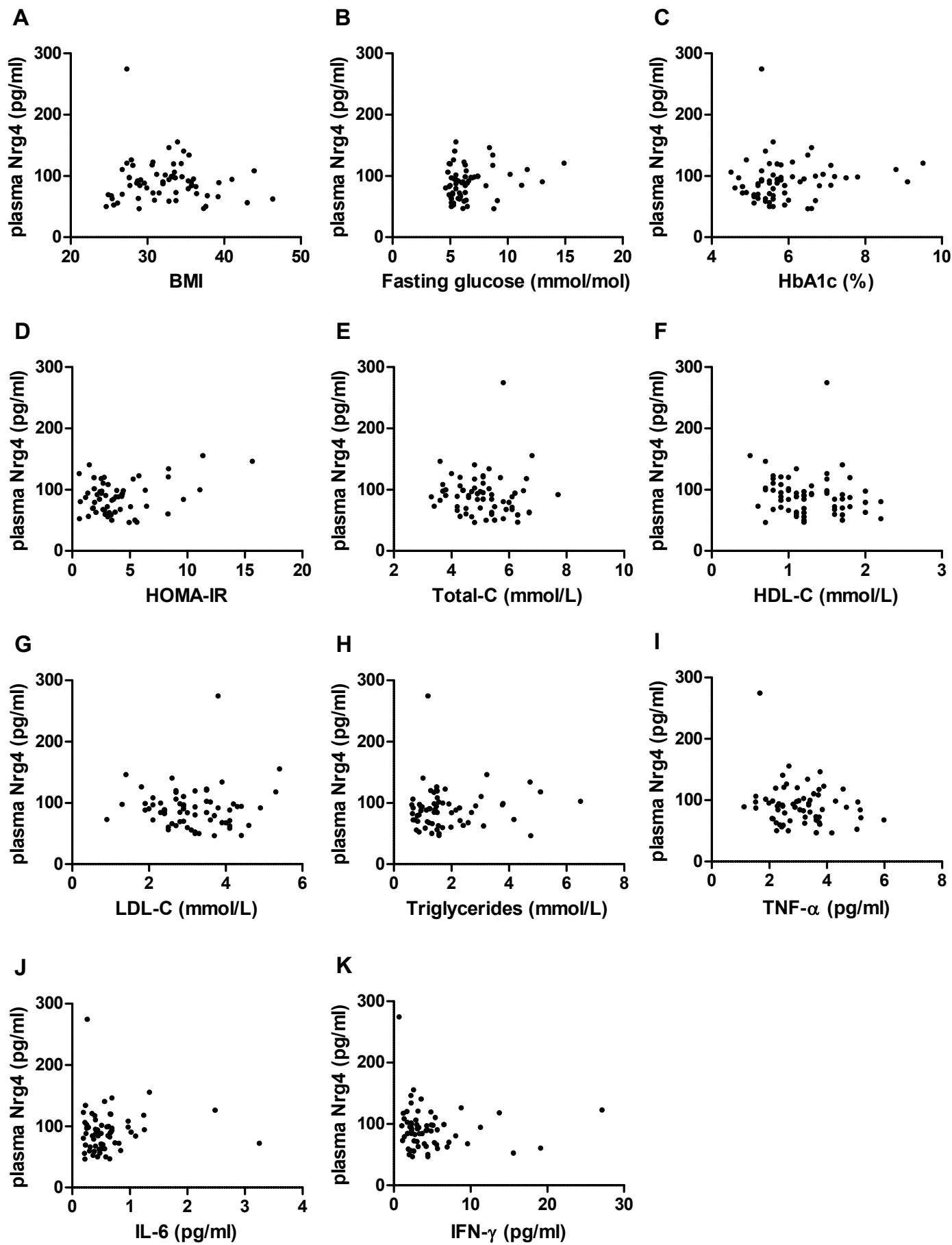

Supplement: S1 Fig — Scatter plot of Nrg4 levels with (A) BMI, (B) Fasting glucose, (C) HbA1C, (D) HOMA-IR, (E) total cholesterol, (F) HDL-C, (G) HDL-C, (H) Triglycerides, (I) TNF-α, (J) IL-6 and (K) IFN-γ. BMI, body mass index; HbA1c, haemoglobin A1c; LDL-C, low-density lipoprotein cholesterol; HDL-C, high-density lipoprotein cholesterol; TNF-α, tumor necrosis factor alpha; IL-6, interleukin 6; IFN-γ, interferon gamma. (PDF) [file pone.0251822.s004.pdf]

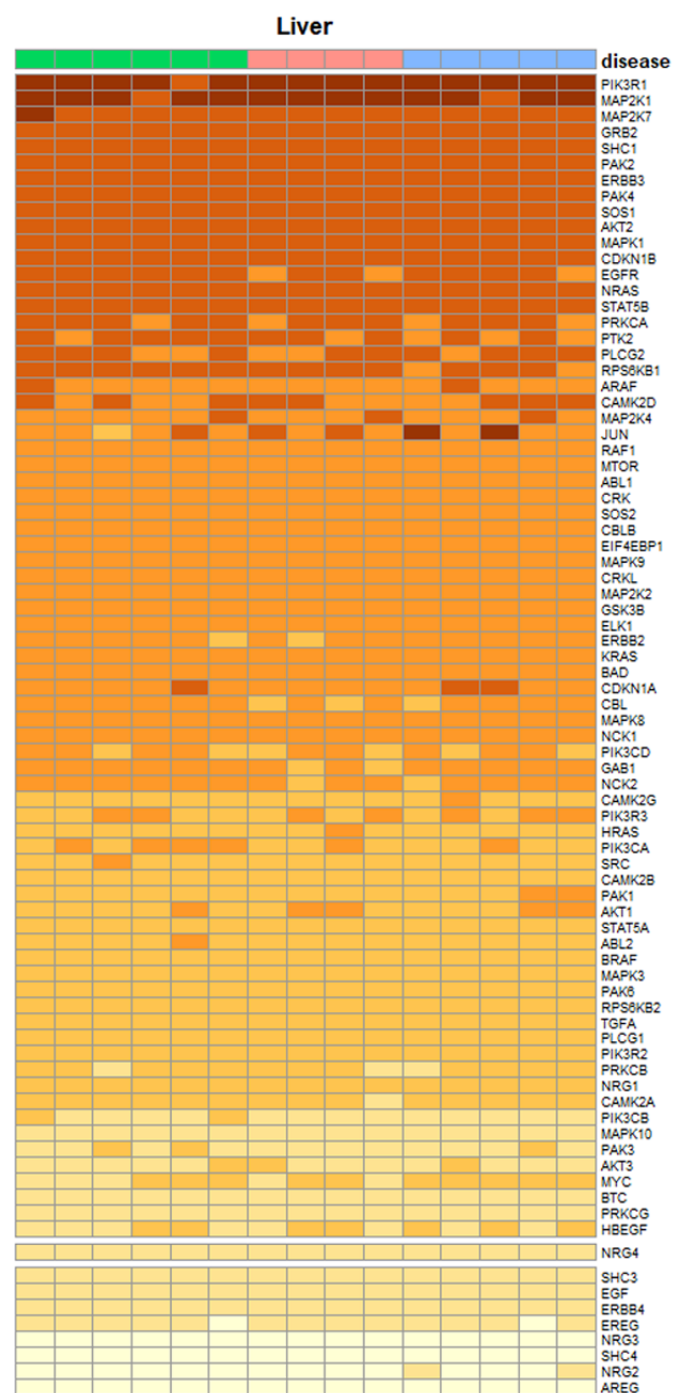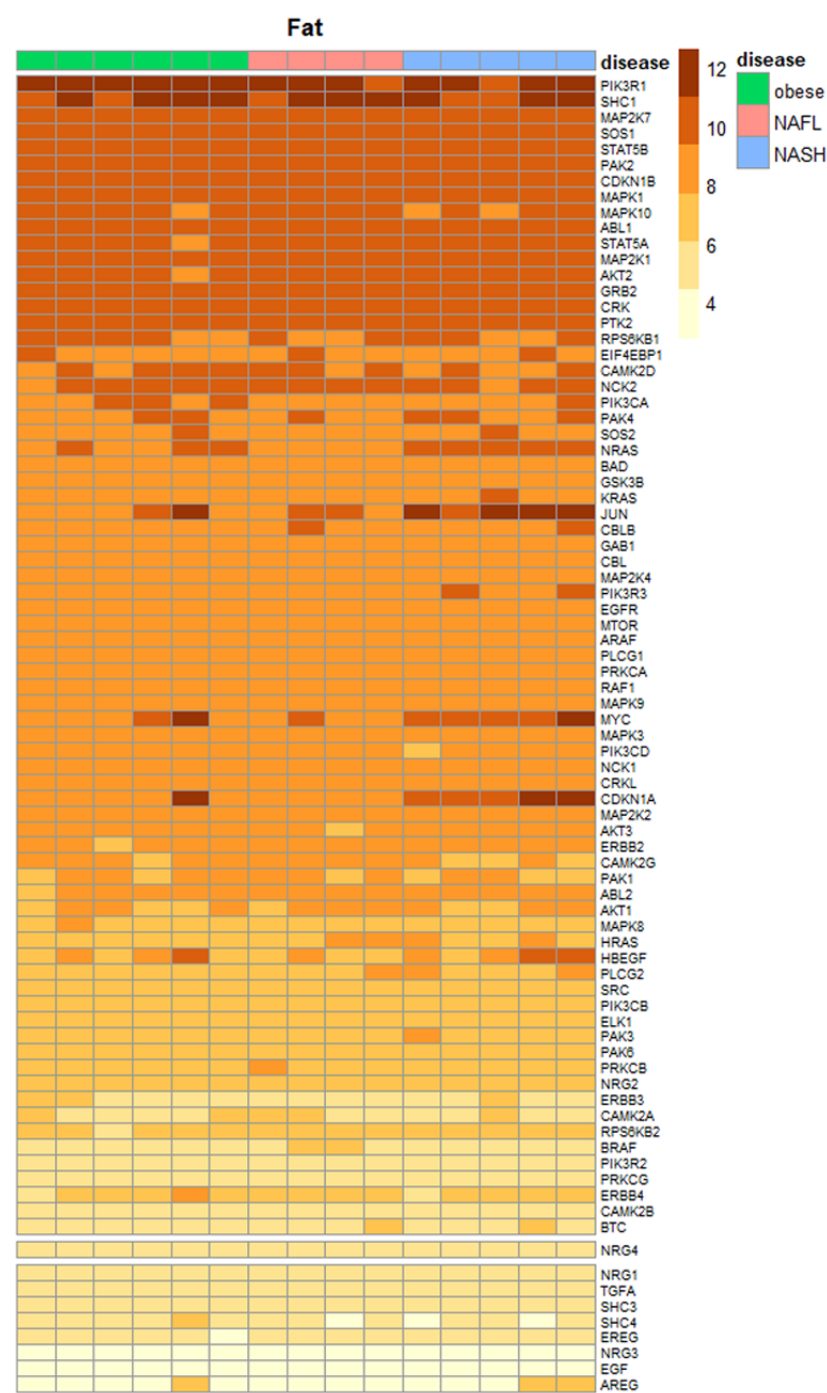

Supplement: S2 Fig — Robust Multi-array (RMA) expressions in the liver and visceral adipose tissue of obese, NAFLD and NASH patients. (PDF) [file pone.0251822.s005.pdf]
